# Significance of Hall effect and Ion slip in a three-dimensional bioconvective Tangent hyperbolic nanofluid flow subject to Arrhenius activation energy

**DOI:** 10.1038/s41598-020-73365-w

**Published:** 2020-10-27

**Authors:** Muhammad Ramzan, Hina Gul, Jae Dong Chung, Seifedine Kadry, Yu-Ming Chu

**Affiliations:** 1 Department of Computer Science, Bahria University, Islamabad, 44000 Pakistan; 2 Department of Mechanical Engineering, Sejong University, Seoul, 143-747 South Korea; 3 Department of Mathematics and Computer Science, Faculty of Science, Beirut Arab University, Beirut, 115020 Lebanon; 4 Department of Mathematics, Huzhou University, Huzhou, 313000 People’s Republic of China; 5 Hunan Provincial Key Laboratory of Mathematical Modeling and Analysis in Engineering, Changsha University of Science and Technology, Changsha, 410114 People’s Republic of China

**Keywords:** Mechanical engineering, Mathematics and computing

## Abstract

The dynamics of partially ionized fluid flow subjected to the magnetic field are altogether distinct in comparison to the flow of natural fluids. Fewer studies are available in the literature discussing the alluring characteristics of the Hall effect and the Ion slip in nanofluid flows. Nevertheless, the flow of nanofluid flow with Hall and Ion slip effect integrated with activation energy, gyrotactic microorganisms, and Cattaneo–Christov heat flux is still scarce. To fill in this gap, our aim here is to examine the three dimensional electrically conducting Tangent hyperbolic bioconvective nanofluid flow with Hall and Ion slip under the influence of magnetic field and heat transmission phenomenon past a stretching sheet. Impacts of Cattaneo–Christov heat flux, Arrhenius activation energy, and chemical reaction are also considered here. For the conversion of a non-linear system to an ordinary one, pertinent transformations procedure is implemented. By using the bvp4c MATLAB function, these equations with the boundary conditions are worked out numerically. The significant impacts of prominent parameters on velocity, temperature, and concentration profiles are investigated through graphical illustrations. The results show that the velocity of the fluid is enhanced once the Ion slip and Hall parameters values are improved. Furthermore, the concentration is improved when the values of the activation energy parameter are enhanced.

## Introduction

The investigation of non-Newtonian liquids is of prime value owing to their industrial and engineering applications. A single constitutive relation can’t be quoted to exhibit the characteristics of such fluids. China clay, soup, toothpaste, shaving creams, blood at a small shear rate, and mayonnaise, etc. are examples of non-Newtonian liquids. Owing to their interesting applications, researchers have described different models of non-Newtonian liquids. Amongst these, the Tangent hyperbolic liquid model is of vital importance that can forecast the shear-thinning phenomenon with marvelous accuracy. The usage of this model in industry and laboratory experiments is recommended by researchers and scientists. Melts, whipped cream, ketchup, and blood are some examples of Tangent hyperbolic fluid. Tangent hyperbolic fluid is a kind of non-Newtonian fluid model which falls in the category of rate type fluids and whose equations are true for both strong and weak shear stresses. In the case of shear-thinning, the rate will dominate the shear stress and vice versa in the case of shear thickening. In 1929, Williamson anticipated a model named “Williamson fluid” with pseudoplastic materials features of shear-thinning and substantiated its results experimentally. Examples of this fluid may include polymer solutions/melts, nail polish, blood, etc. However, the Tangent hyperbolic fluid model has an edge over its contemporary models owing to its unique features of computational comfort, simplicity, and physical robustness. Shafiq et al.^1^ found an analytical solution of Tangent hyperbolic bio-convective nanomaterial flow holding motile microorganisms by using the Homotopy Analysis Method (HAM). They perceived that for strong magnetic effects, the velocity profile reduces. They also witnessed an opposite behavior of concentration and temperature profiles for mounting estimates of the thermophoresis parameter. Hayat et al.^2^ explored MHD Tangent hyperbolic nanomaterial flow with variable thickness. From this study, it is comprehended that velocity is a decreased for growing estimations of the Weissenberg number and magnetic parameter. Ibrahim and Gizewu^3^, addressed the Tangent hyperbolic nanofluid flow with double diffusion effects and a second-order slip condition at the boundary. Ramzan et al.^4^ discussed the second-order slip in the attendance of Cattaneo–Christov (C–C) heat flux and homogeneous–heterogeneous reactions with Tangent hyperbolic liquid flow. The flow behavior of Tangent hyperbolic liquid (blood) with copper nanoparticles over a ciliated tube is studied by Maqbool et al.^5^. Some more recent investigations highlighting various aspects of Tangent hyperbolic fluid flow may be seen at Refs.^6–9^ and many therein.

Arrhenius activation energy is the least essential energy mandatory to initiate a chemical reaction. It is coined by Arrhenius in 1889. Activation energy plays the role of an obstacle between un-reacted and reacted molecules or atoms. Once this barrier is crossed, the chemical reaction will occur, and, in such situations, only those molecules or atoms will cross the barricade that has more energy than the fence. Awad et al.^10^ analyzed the impacts of activation energy and binary chemical reaction in a viscous rotating flow utilizing the Spectral relaxation method (SRM). It is revealed that concentration distribution is increased once the values of dimensionless activation energy are boosted. It is also noticed that the velocity field is monotonically decayed versus a feeble fluid rotation rate. Lu et al.^11^ studied numerically a 3D nano liquid flow past a moving elongated plate with combined impacts of Arrhenius activation energy and binary chemical reaction. The proposed mathematical model is also accompanied by gyrotactic microorganisms, Joule heating, thermal radiation with anisotropic slip at the boundary. It is disclosed that microorganism’s local density declines versus nondimensional activation energy. The flow of second-grade nano liquid flow with the Arrhenius activation energy and binary chemical reaction in a spongy media is studied numerically by Khan et al.^12^. The flow analysis also caters to the behaviors of thermal radiation and Entropy generation. It is stated that the concentration is declined for large values of chemical reaction and activation parameters. Koriko et al.^13^ studied the flow of Micropolar fluid with Exothermic and Endothermic chemical reactions past a vertical permeable stretched surface. Some recent attempts discussing activation energy may be found at Refs.^11,14–16^.

The convective movement of fluid at the microscopic level owing to the density gradient is termed as Bio-convection and is generated by the swimming of motile organisms. The density of the customary fluid is enhanced by self-driven microorganisms in a specific direction thus generating bio-convection. A good number of applications in wastewater, agriculture, soil, and microbial engineering may be found in the real world associated with bioconvective microorganisms. The idea of bioconvective nanofluid was coined by Kuznetsov^17,18^. Kuznetsov^19^ focused to visualize the effect of nano-liquid with gyrotactic microorganisms. Aziz et al.^20^ examined the steady boundary layer flow in a porous medium with an amalgamation of gyrotactic microorganisms and nano-liquid. The behavior of bioconvective magnetic field, thermophoresis, and Brownian motion on a free convection nano-liquid flow comprising the gyrotactic microorganisms over an elongating sheet is explored by Akbar and Khan^21^. Alsaedi et al.^22^ introduced the effect of nanofluid with gyrotactic microorganisms. Lately, Khan et al.^23^ deliberated the effect of generalized Burgers liquid flow with chemical reactions and magneto nanoparticle. The variable thermophysical effects on bioconvective nano-liquid are examined by Begum et al.^24^. More studies highlighting the role of bio-convection are appended at Refs.^11,25–29^.

In all the above-quoted works, the impacts of Hall and Ion slip are not considered in employing the ohm’s law as there is no significant effect for the tenuous magnetic field. Nevertheless, nowadays applications of magnetohydrodynamics are preferred with the strong applied magnetic field so that the impact of magnetohydrodynamics is perceptible. That is why the Hall and Ion slip are vital and they have significant effects on the magnitude, the current density’s direction, and subsequently on the magnetic force term^30^. The behavior of ionized fluids under the effect of the magnetic field has entirely different traits in comparison to the non-ionized fluids. The ionized fluids are influenced by three major factors named (i) the magnetic force owing to an applied magnetic field, (ii) the Hall force, because of electrons’ collision, (iii) the Ion slip force, owing to ions’ collision. Most of the existing works state the flows influenced by the Lorentz force. Fewer studies may be found with Hall's current effects. But the literature highlighting the impacts of both Ion and hall slip is scarce. Abdelsalam and Bhatti^31^ studied the Tangent hyperbolic nanofluid peristaltic flow influenced by chemical reaction and Ion and Hall slip impacts in a non-uniform channel. The prime outcome of the study is that the chemical reaction depicts dual solutions for the concentration profile for higher estimates of the Brownian motion parameter. The flow of nanofluid comprising varied metallic particles and their oxides like copper, silver, and aluminum oxide are immersed into the blood with Ion and Hall slip effects amalgamated with heat generation/absorption is deliberated by Nawaz et al.^32^. It is revealed that the application of Ion and Hall slip results in a substantial decrease in heat dissipation in the presence applied magnetic field. A comparative analysis of two types of nanofluid flows namely 36 nm Al_2_O_3_-water and 47 nm Al_2_O_3_-water over an upper horizontal surface of a paraboloid is analyzed in the attendance of Hall effect by Animasaun et al.^33^. It is observed in this study that cross fluid velocity in the case of 36 nm nanoparticles is dominant in comparison to the 47 nm nanoparticles. Recent studies focusing on Hall and Ion slip effect may be found at Refs.^34,35^.

In the present era, the alluring characteristics of the nanofluids have attracted researchers and scientists to dig out new avenues. The nanofluid is an engineered fluid comprising an amalgamation of metallic particles or their associated oxides and some customary liquid like water. The heat transfer capabilities of nanofluids are amazingly much higher than those of the routine liquids. This tempting feature of nanofluids is so captivating that it has revolutionized the modern technological world. One can find its applications from the cooling of microchips to atomic reactors. The latest studies emphasizing various attributes of nanofluids may be found in Refs.^36–43^. Additionally, copious applications under the impact of the magnetic field may be found in engineering, medicine, and physics. Fluids under magnetic field influence have interesting applications like MHD generators, treatment of tumors, and pumps, etc. The strength of the applied magnetic field and arrangements of molecules highly affects the fluids’ behavior. The application of the magnetic field alters the arrangement of nanoparticles and eventually the concentration of the fluid. A nanofluid with magnetic physiognomies is termed as magnetic nanofluid with vast range applications including optical modulators and switches. A good number of magneto-nanofluids explorations may be found in the literature^44–46^.

The aforesaid literature review reveals that very few explorations are discussed that signifies the partially ionized nanofluid flows. However, no study so far carried out that communicates the activation energy amalgamated in a partially ionized Tangent hyperbolic nanofluid flow under the influence of magnetohydrodynamics. The additional attributes of Cattaneo–Christov heat flux with convective boundary condition in the envisioned mathematical model also distinguish the present study from the existing literature. The consideration of these impacts turns the problem into a highly nonlinear system. The numerical solution of the problem is uncovered by using the Runge–Kutta-4 technique with the shooting method. The manuscript is structured in five sections. The modeling is of the envisioned problem is given in “Mathematical formulation”. The numerical solution method is presented in “Numerical computations”. “Results and discussion” is devoted to graphical and numerical results’ discussion. The concluding comments emphasizing key results are presented in the last section.

While studying this exploration, we will try to answer the subsequent questions:

i. What is the effect of Hall and Ion slip on the fluid velocity?
ii. What is the role of activation energy in this model?
iii. How “bioconvection” affect the motile density profile?
iv. Does the temperature of the fluid is influenced by the magnetic field effect?
v. How Cattaneo–Christov heat flux contributes to the nanofluid flow?

## Mathematical formulation

**Figure 1:**
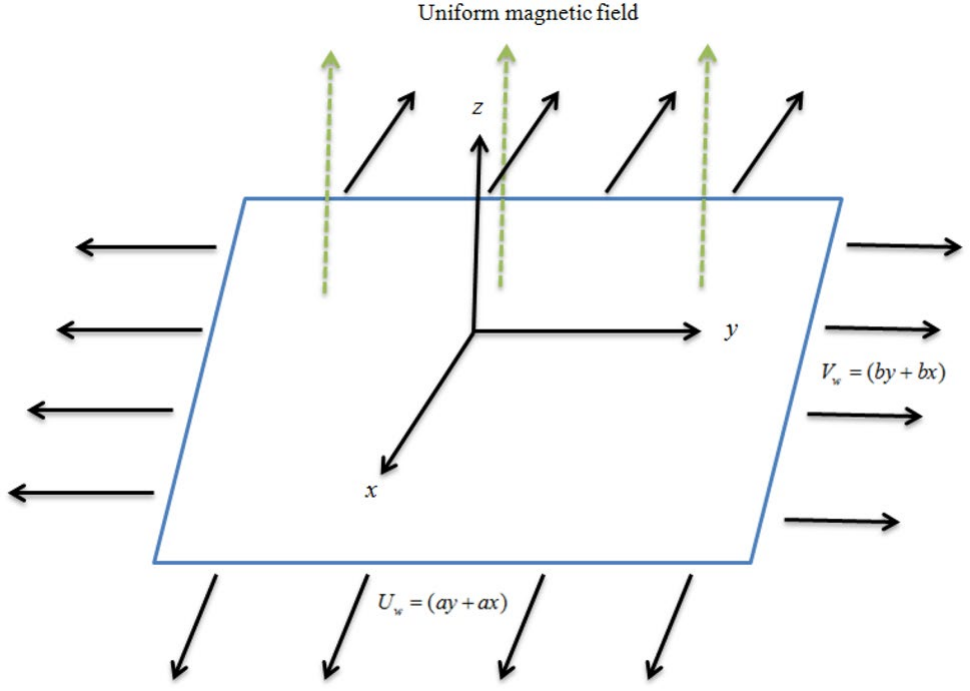
Flow geometry.

Let us assume heat transfer in partially ionized 3D tangent hyperbolic nano liquid past a surface with velocity $V_{w} = [(x + y)a,(y + x)b]$**,** Conversion of activation energy, Chemical reaction, bioconvective gyrotactic organisms with CC heat flux and Ohmic dissipation likewise consider (Fig. 1).

The boundary layer governing equations depicting the assumed model is^11,31,47^:

1 $$ u_{x} + v_{y} + w_{z} = 0, $$

2 $$ uu_{x} + vu_{y} + wu_{z} = (1 - n)\nu u_{zz} + n\sqrt 2 \nu \Gamma u_{z} u_{zz} + \frac{{\sigma B_{0}^{2} }}{{\rho [\beta_{e}^{2} + (1 + \beta_{e} \beta_{i} )^{2} ]}}\left[ {\beta_{e} v - (1 + \beta_{e} \beta_{i} )u} \right] - \frac{\nu }{K}u - Fu^{2} , $$

3 $$ uv_{x} + vv_{y} + wv_{z} = (1 - n)\nu v_{zz} + n\sqrt 2 \nu \Gamma v_{z} v_{zz} - \frac{{\sigma B_{0}^{2} }}{{\rho [\beta_{e}^{2} + (1 + \beta_{e} \beta_{i} )^{2} ]}}\left[ {\beta_{e} u - (1 + \beta_{e} \beta_{i} )v} \right] - \frac{\nu }{K}v - Fv^{2} , $$

4 $$ \begin{aligned} & u\mathop T\limits^{ * }_{x} + v\mathop T\limits^{ * }_{y} + w\mathop T\limits^{ * }_{z} = \frac{k}{{\rho c_{p} }}\mathop T\limits^{ * }_{zz} - \lambda_{2} \left( \begin{gathered} u^{2} \mathop T\limits^{ * }_{xx} + v^{2} \mathop T\limits^{ * }_{yy} + w^{2} \mathop T\limits^{ * }_{zz} + (uu_{x} + vu_{y} + wu_{z} )\mathop T\limits^{ * }_{x} \hfill \\ + (uv_{x} + vv_{y} + wv_{z} )\mathop T\limits^{ * }_{y} \hfill \\ + (uw_{x} + vw_{y} + ww_{z} )\mathop T\limits^{ * }_{z} + 2uv\mathop T\limits^{ * }_{xy} + 2vw\mathop T\limits^{ * }_{yz} + 2uw\mathop T\limits^{ * }_{xz} \hfill \\ \end{gathered} \right) \\ & \quad + \frac{{Q_{0} }}{{\rho c_{p} }}\left(\mathop T\limits^{ * } - \mathop T\limits^{ * }_{\infty } \right) + \tau D_{B} C_{z} \mathop T\limits^{ * }_{z} + \tau \frac{{D_{T} }}{{T_{\infty } }}\mathop T\limits^{ * } \,_{z}^{2} + \frac{{\sigma B_{0}^{2} }}{{\rho [\beta_{e}^{2} + (1 + \beta_{e} \beta_{i} )^{2} ]}}[u^{2} + v^{2} ], \\ \end{aligned} $$

5 $$ uC_{x} + vC_{y} + wC_{z} = D_{B} C_{zz} + \frac{{D_{T} }}{{\mathop T\limits^{*}_{\infty } }}\mathop T\limits^{*}_{zz} - K_{c}^{2} (C - C_{\infty } )\left( {\frac{{\mathop T\limits^{*} }}{{\mathop T\limits^{*}_{\infty } }}} \right)^{n} e^{{ - \frac{{E_{a} }}{{k\mathop T\limits^{*} }}}} , $$

6 $$ uN_{x} + vN_{y} + wN_{z} + \frac{{bW_{c} }}{{\left( {C_{w} - C_{\infty } } \right)}}\left[ {N_{z} C_{z} + NC_{zz} } \right] = D_{n} N_{zz} , $$

with associated suitable boundary conditions:

7 $$ \begin{gathered} u = U_{w} = (y + x)a,\,\,v = V_{w} = (y + x)b,\,\,w = 0,\,\, - k\mathop T\limits^{*}_{z} = h_{f} (\mathop T\limits^{*}_{w} - \mathop T\limits^{*} ),\,\,C = C_{w} + \alpha C_{z} ,\,\,N = N_{w} ,\,{\text{at}}\,z = 0 \hfill \\ u \to 0,\,\,\,\,\,v \to 0,\,\,\,\mathop T\limits^{*} = \mathop T\limits^{*}_{\infty } ,\,\,\,\,\,C = C_{\infty } ,N = N_{\infty } ,{\text{as}}\,z \to \infty . \hfill \\ \end{gathered} $$

Similarity transformations are defined as:

8 $$ \begin{aligned} & u = (x + y)af^{\prime},\,\,v = (x + y)ag^{\prime},\,\,w = - (g + f)\sqrt {av} , \\ & \theta = \frac{{\mathop T\limits^{*} - \mathop T\limits^{*}_{\infty } }}{{\mathop T\limits^{*}_{w} - \mathop T\limits^{*}_{\infty } }},\,\,\varphi = \frac{{C - C_{\infty } }}{{C_{w} - C_{\infty } }},\,\,\eta = \sqrt {\frac{a}{\nu }} z,\,\,\xi = \frac{{N - N_{\infty } }}{{N_{w} - N_{\infty } }}. \\ \end{aligned} $$

Using Eq. (8), Eqs. (2)–(7) become:

9 $$ \begin{aligned} & (1 - n)f^{\prime\prime\prime} + nWef^{\prime\prime}f^{\prime\prime\prime} - \lambda *f^{\prime} - F_{r} f^{\prime 2} + (g + f)f^{\prime\prime} - f^{\prime 2} - g^{\prime}f^{\prime} \\ & \quad + \frac{{M^{2} }}{{\beta_{e}^{2} + (1 + \beta_{e} \beta_{i} )^{2} }}\left[ {g^{\prime}\beta_{e} - f^{\prime}(1 + \beta_{i} \beta_{e} )} \right] = 0, \\ \end{aligned} $$

10 $$ \begin{aligned} & (1 - n)g^{\prime\prime\prime} + nWeg^{\prime\prime}g^{\prime\prime\prime} + (f + g)g^{\prime\prime} - g^{\prime 2} - g^{\prime}f^{\prime} - \lambda *g^{\prime} - F_{r} g^{\prime 2} \\ & \quad - \frac{{M^{2} }}{{\beta_{e}^{2} + (1 + \beta_{e} \beta_{i} )^{2} }}\left[ {f^{\prime}\beta_{e} + g^{\prime}(1 + \beta_{e} \beta_{i} )} \right] = 0, \\ \end{aligned} $$

11 $$ \begin{aligned} & \frac{1}{\Pr }\theta^{\prime\prime} - \gamma \left[ {(g + f)(f^{\prime} + g^{\prime})\theta ^{\prime} + (g + f)^{2} \theta^{\prime\prime}} \right] + Q\theta + N_{b} \theta^{\prime}\varphi^{\prime} + N_{t} \theta^{\prime 2} \\ & \quad + \frac{{M^{2} Ec}}{{\beta_{e}^{2} + (1 + \beta_{e} \beta_{i} )^{2} }}(g^{\prime 2} + f^{\prime 2} ) + (g + f)\theta \prime = 0, \\ \end{aligned} $$

12 $$ \varphi^{\prime\prime} + Sc(g + f)\varphi^{\prime} + \frac{{N_{t} }}{{N_{b} }}\theta^{\prime\prime} - K_{r} Sc\varphi (1 + \delta \theta )^{n} e^{{\frac{ - E}{{(1 + \delta \theta )}}}} = 0. $$

13 $$ \xi^{\prime\prime} + Lb(g + f)\xi^{\prime} - Pe[(\Omega + \xi )\varphi^{\prime\prime} + \xi^{\prime}\varphi^{\prime}] = 0, $$

with associated boundary conditions

14 $$ \begin{gathered} f(0) = 0,\,\,f^{\prime}(0) = 1,\,\,g(0) = 0,\,\,g^{\prime}(0) = \lambda ,\,\,\theta^{\prime} = - Bi(1 - \theta ),\varphi (0) = 1 + B\varphi^{\prime}(0),\,\xi (0) = 1, \hfill \\ f^{\prime}(\infty ) = 0,\,\,g^{\prime}(\infty ) = 0,\,\,\theta (\infty ) = 0,\,\,\varphi (\infty ) = 0,\xi (\infty ) = 0. \hfill \\ \end{gathered} $$

The above quantities are defined as

15 $$ \begin{aligned} & \beta_{e} = \omega_{e} \tau_{e} ,\,\,M^{2} = \frac{{\sigma B_{0}^{2} }}{\rho a},\,\,\beta_{i} = \omega_{i} \tau_{i} ,\Pr = \frac{{\mu c_{p} }}{k},\,\,Ec = \frac{{U_{w}^{2} }}{{c_{p} (T_{w} - T_{\infty } )}},\lambda = \frac{b}{a}, \\ & \gamma = \lambda_{2} a,\,\,Q = \frac{{Q_{0} }}{{\rho c_{p} a}},\,\,\delta = \frac{\Delta T}{{T_{\infty } }},\,\,N_{b} = \frac{{\tau D_{B} C_{\infty } }}{\nu },\,\,N_{t} = \frac{{\tau D_{T} \Delta T}}{{T_{\infty } \nu }},E = \frac{Ea}{{kT_{\infty } }}, \\ & K_{r} = \frac{{K_{c}^{2} }}{a},\,\,Bi = \frac{{h_{f} }}{k}\sqrt {\frac{\nu }{a}} ,\,\,We = \frac{{\sqrt {2a} \Gamma U_{w} }}{\sqrt \nu },\,\,Lb = \frac{v}{{D_{m} }},\,\,\,\Omega = \frac{{n_{\infty } }}{{n_{f} - n_{\infty } }}, \\ & \beta_{e} = \omega_{e} \tau_{e} ,\,\,M^{2} = \frac{{\sigma B_{0}^{2} }}{\rho a},\,\,\beta_{i} = \omega_{i} \tau_{i} ,Pe = \frac{{bW_{c} }}{{D_{m} }}. \\ \\ \end{aligned} $$

Drag force coefficient in $C_{fx}$ and $C_{gy}$ , local Sherwood number and local density of the number of the motile microorganisms are defined by:

16 $$ C_{fX} = \frac{{\left. {\tau_{zX} } \right|_{z = 0} }}{{\rho a^{2} (x + y)^{2} }},\,\,\,\,\,\,C_{gY} = \frac{{\left. {\tau_{zY} } \right|_{z = 0} }}{{\rho a^{2} (x + y)^{2} }},\,Sh_{X} = \left. {\frac{{(x + y)q_{m} }}{{D_{B} (C_{w} - C_{\infty } )}}} \right|_{z = 0} ,Nn_{X} = \frac{{(x + y)q_{n} }}{{D_{n} \left( {n_{f} - n_{{_{\infty } }} } \right)}}, $$

where

17 $$ \begin{aligned} & \tau_{zX} = [(1 - n)u_{z} + \frac{n\Gamma }{{\sqrt 2 }}u_{z}^{2} ]_{z = 0} ,\,\,\,\,\,\,\tau_{zY} = [(1 - n)v_{z} + \frac{n\Gamma }{{\sqrt 2 }}v_{z}^{2} ]_{z = 0} , \hfill \\ & q_{m} = \left. { - D_{B} C_{z} } \right|_{z = 0} ,q_{n} = - D_{n} \left. {n_{z} } \right|_{z = 0} . \hfill \\ \end{aligned} $$

The dimensionless Skin friction coefficients, local Sherwood number, and local density number of the motile microorganisms are appended as follows:

18 $$ \begin{gathered} C_{fX} \sqrt {{\text{Re}}_{X} } = (1 - n)f^{\prime\prime}(0) + \frac{n}{2}We\left( {f^{\prime\prime}(0)} \right)^{2} , \hfill \\ C_{gY} \sqrt {{\text{Re}}_{X} } = (1 - n)g^{\prime\prime}(0) + \frac{n}{2}We\left( {g^{\prime\prime}(0)} \right)^{2} , \hfill \\ Sh_{X} {\text{Re}}^{ - 0.5} = - \varphi ^{\prime}\left( 0 \right),Nn_{X} {\text{Re}}_{X}^{ - 1/2} = - \xi ^{\prime}\left( 0 \right). \hfill \\ \end{gathered} $$

With local Reynolds number

19 $$ {\text{Re}} = \frac{{U_{w}^{2} }}{a\nu }. $$

## Numerical computations

The system of equations defined above is handled by the MATLAB software bvp4c tool. The infinite domain is taken as $\eta = 4$ which is sufficient to examine the asymptotic execution of the solution. The initial approximation with a tolerance 10^–6^ is acceptable to define a numerical solution. The initial conditions equivalent to the boundary conditions must meet the solution system. In this direction, First of all, new variables are introduced as:

20 $$ \begin{aligned} & f = Y_{1} ,f^{\prime} = Y_{2} ,f^{\prime\prime} = Y_{3} ,f^{\prime\prime\prime} = Y_{3}^{^{\prime}} ,g = Y_{4} ,g^{\prime} = Y_{5} ,g^{\prime\prime} = Y_{6} , \\ & g^{\prime\prime\prime} = y_{6}^{^{\prime}} ,\theta = y_{7} ,\theta^{\prime} = y_{8} ,\theta^{\prime\prime} = y^{\prime}_{8} ,\varphi = y_{9} ,\varphi^{\prime} = y_{10} ,\varphi^{\prime\prime} = Y_{10}^{^{\prime}} , \\ & \xi = Y_{11} ,\xi^{\prime} = Y_{12} ,\xi^{\prime\prime} = Y_{12}^{^{\prime}} . \\ \end{aligned} $$

Using the above expressions in MATLAB bvp4c we have the following set of first-order equations:

21 $$ \begin{aligned} & Y_{3}^{^{\prime}} = [(1 - n)/nWey_{3} [(Y_{4} + Y_{1} )Y_{3} + \lambda *Y_{2} + F_{r} Y_{2}^{2} + Y_{2}^{2} + Y_{5} Y_{2} \\ & \quad - \frac{{M^{2} }}{{\beta_{e}^{2} + (1 + \beta_{e} \beta_{i} )^{2} }}(Y_{5} \beta_{e} - Y_{2} (1 + \beta_{i} \beta_{e} )], \\ \end{aligned} $$

22 $$ \begin{aligned} & Y_{6}^{^{\prime}} = [(1 - n)/nWeY_{6} ][(Y_{4} + Y_{1} )Y_{6} + \lambda *Y_{5} + F_{r} Y_{5}^{2} + Y_{5}^{2} + Y_{5} Y_{2} \\ & \quad + \frac{{M^{2} }}{{\beta_{e}^{2} + (1 + \beta_{e} \beta_{i} )^{2} }}(Y_{2} \beta_{e} + Y_{5} (1 + \beta_{i} \beta_{e} )], \\ \end{aligned} $$

23 $$ \begin{aligned} & Y_{8}^{^{\prime}} = [(Y_{4} + Y_{1} )(Y_{2} + Y_{5} )Y_{8} - QY_{7} + Y_{2}^{2} + Y_{5} Y_{2} - \frac{{M^{2} }}{{\beta_{e}^{2} + (1 + \beta_{e} \beta_{i} )^{2} }}(Y_{5} \beta_{e} - Y_{2} (1 + \beta_{i} \beta_{e} )) \\ & \quad - N_{b} Y_{8} Y_{10} - N_{t} Y_{8}^{2} - \frac{{M^{2} Ec}}{{\beta_{e}^{2} + (1 + \beta_{e} \beta_{i} )^{2} }}(Y_{5}^{2} + Y_{2}^{2} ) - (Y_{1} + Y_{4} )Y_{8} ]/\left[ {\frac{1}{\Pr } - \lambda_{1} (Y_{4} + Y_{1} )} \right], \\ \end{aligned} $$

24 $$ Y^{\prime}_{10} = - Sc(Y_{1} + Y_{4} )Y_{10} - \frac{{N_{t} }}{{N_{b} }}Y^{\prime}_{8} + K_{r} ScY_{9} (1 + \delta Y_{7} )^{n} e^{{\frac{ - E}{{(1 + \delta Y_{7} )}}}} , $$

25 $$ Y^{\prime}_{12} = - Lb(Y_{4} + Y_{1} )\xi^{\prime} + Pe[(\Omega + \xi )Y_{10}^{^{\prime}} + \xi^{\prime}Y_{9} ], $$

with the transformed BCs

26 $$ \begin{aligned} &Y_{1} (0) = Y_{2} (\infty ) = 0,Y_{2} (0) = 1,Y_{4} (0) = Y_{5} (\infty ) = 0,Y_{5} (0) = \lambda ,Y_{7} (0) = - Bi(1 - Y_{8} (0), \hfill \\ & Y_{9} (0) = 1 + BY_{10} (0),Y_{11} (0) = 1;Y_{7} (\infty ) = 0,Y_{9} (\infty ) = 0,Y_{11} (\infty ) = 0. \hfill \\ \end{aligned} $$

## Results and discussion

**Figure 2:**
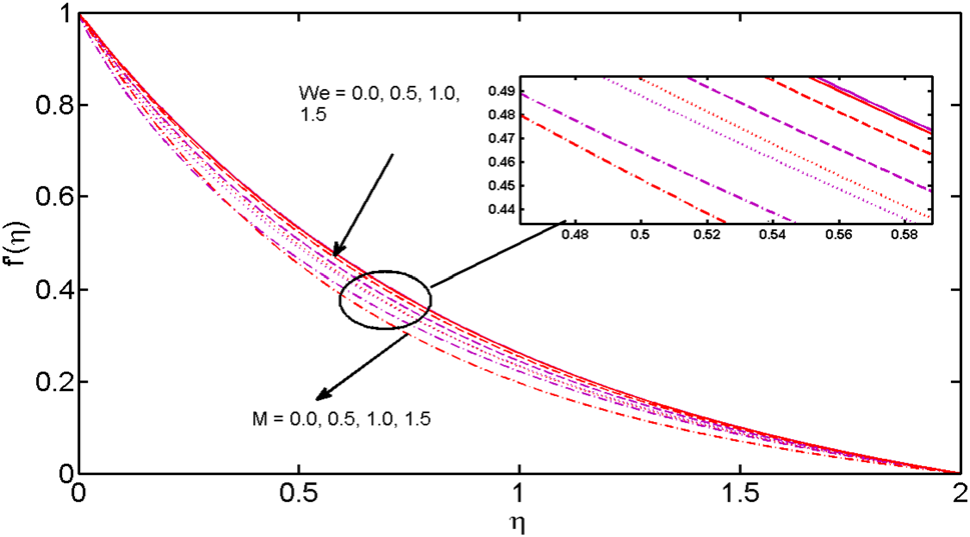
$M$ and $We$ variations vs. $f^{\prime}(\eta )$.

**Figure 3:**
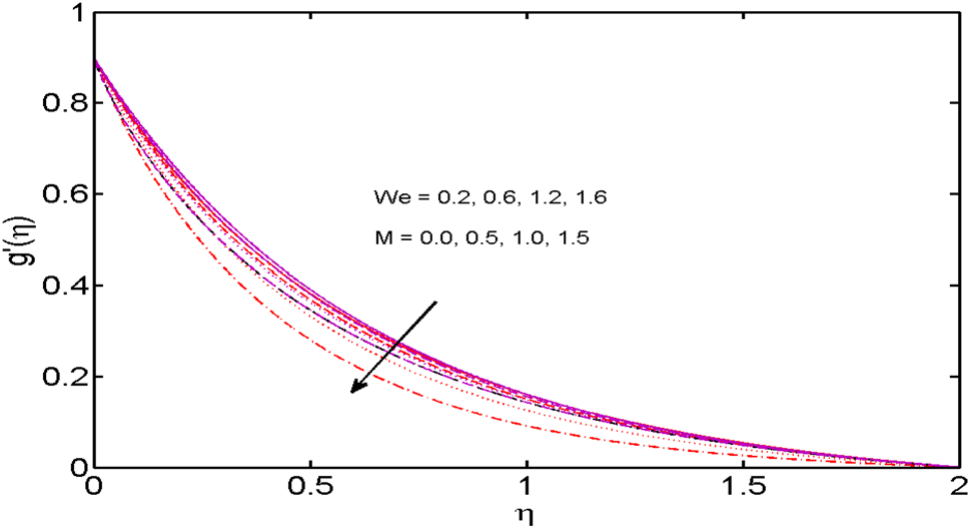
$M$ and $We$ variations vs. $g^{\prime}(\eta )$

**Figure 4:**
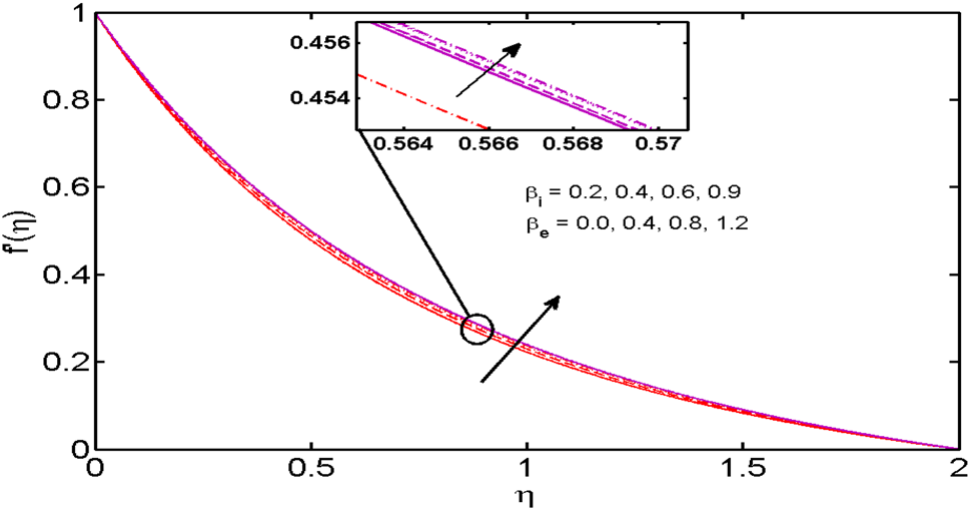
$\beta_{i}$ and $\beta_{e}$ variations vs. $f^{\prime}(\eta )$.

**Figure 5:**
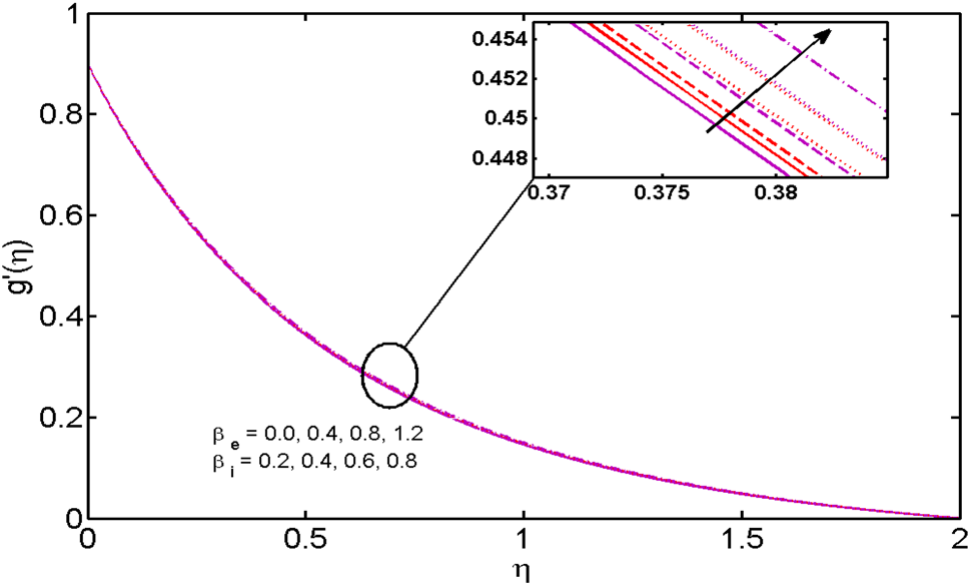
$\beta_{i}$ and $\beta_{e}$ variations vs. $g^{\prime}(\eta )$.

**Figure 6:**
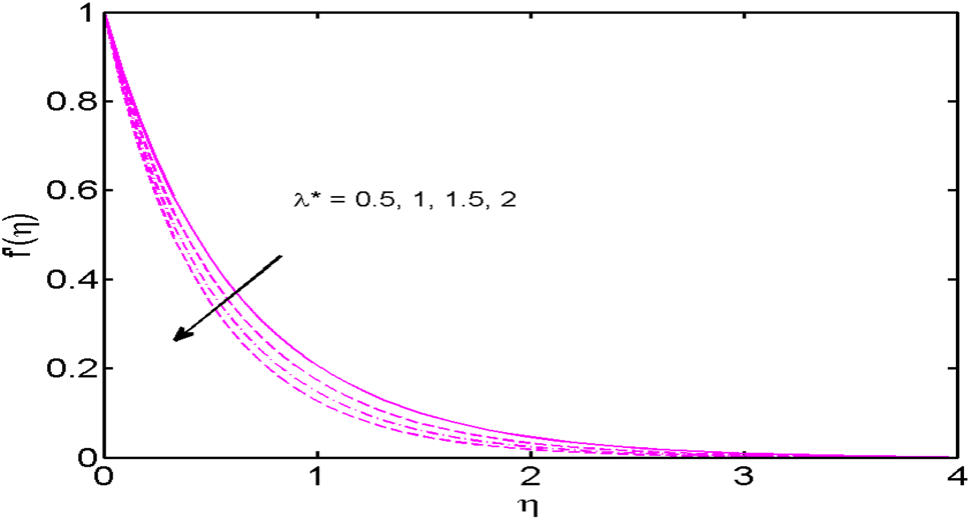
$$\lambda *$$ variations vs. $$f^{\prime}(\eta )$$.

**Figure 7:**
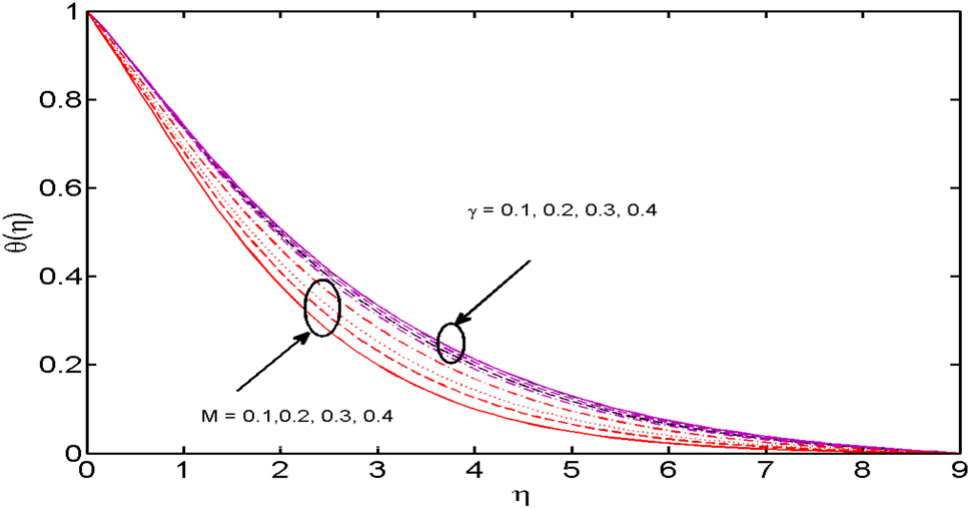
$M$ and $\gamma$ variations vs. $\theta (\eta )$.

**Figure 8:**
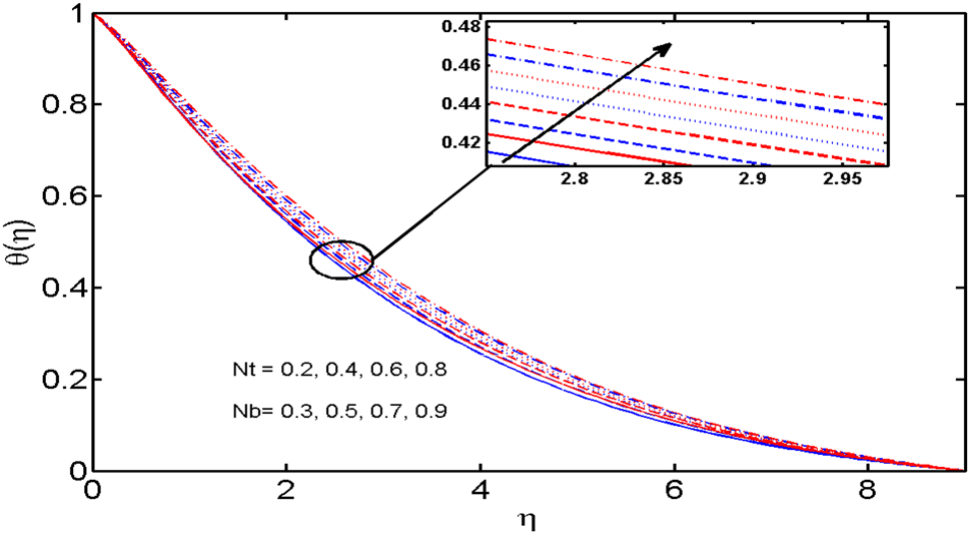
$N_{t}$ and $N_{b}$ variations vs. $\theta (\eta )$.

**Figure 9:**
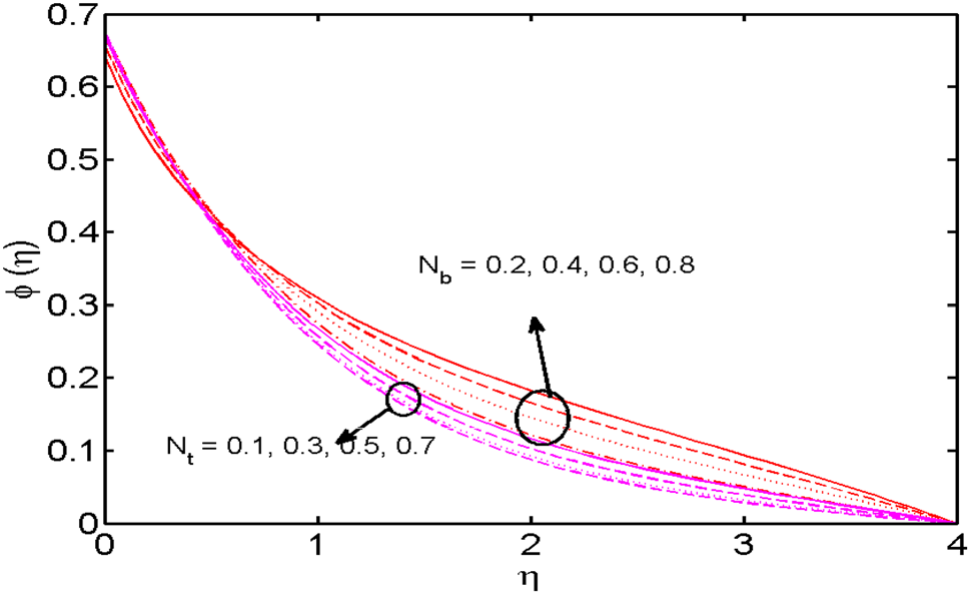
$N_{t}$ and $N_{b}$ variations vs. $\phi (\eta )$.

**Figure 10:**
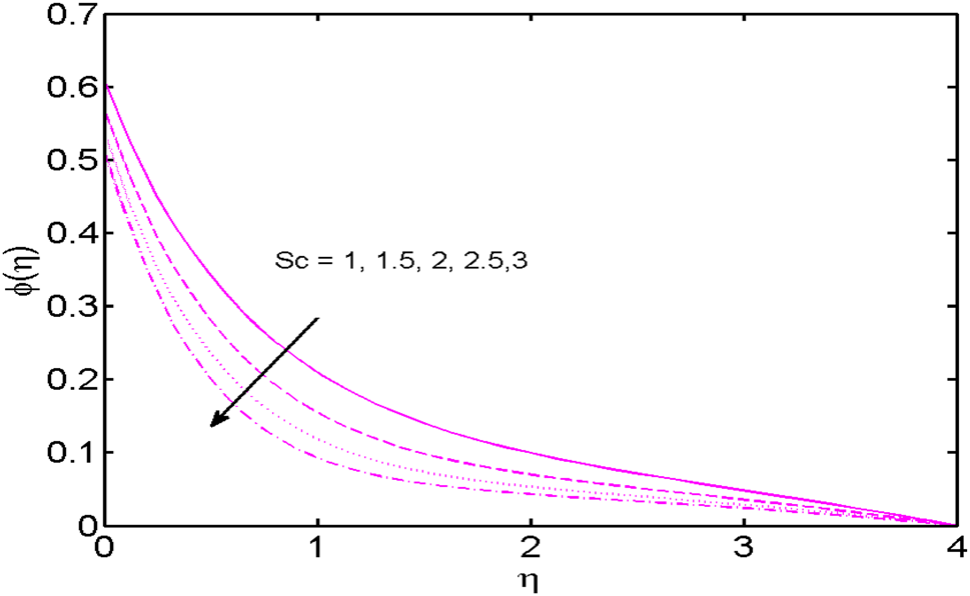
$$Sc$$ variations vs. $$\varphi (\eta )$$.

**Figure 11:**
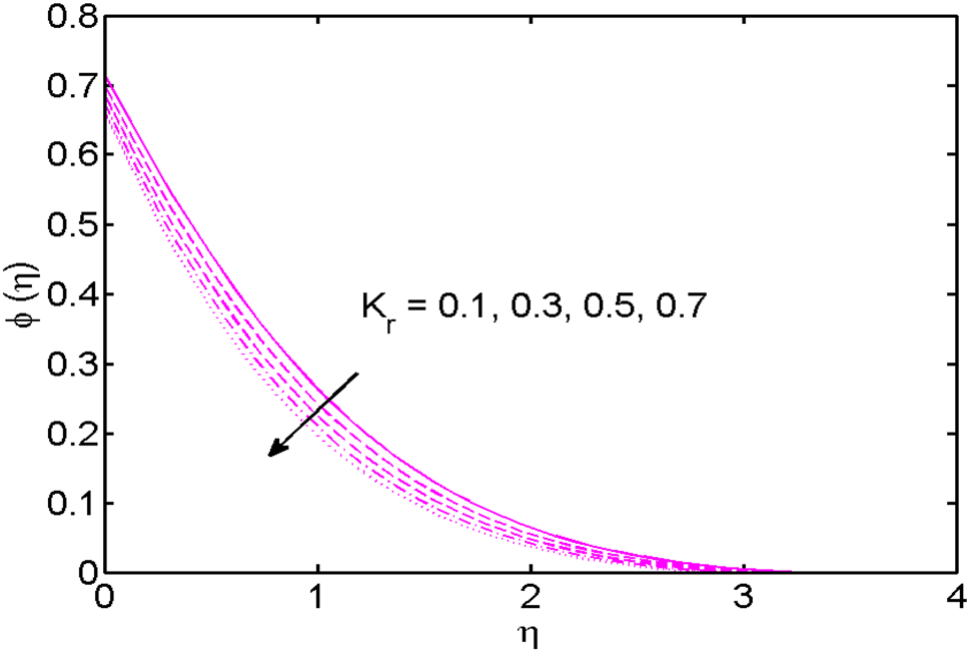
$$K_{r}$$ variations vs. $$\varphi (\eta )$$.

**Figure 12:**
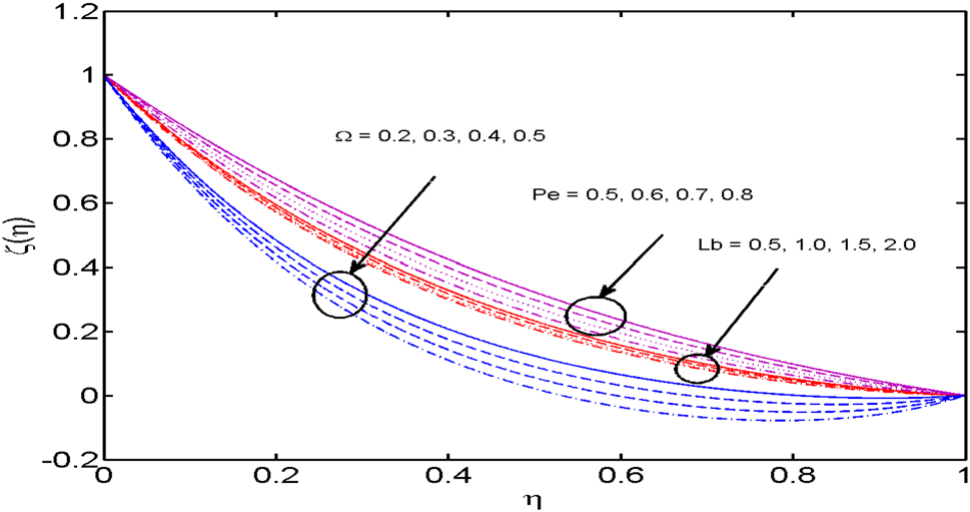
$\Omega ,P_{e}$ and $L_{b}$ variations vs. $\xi (\eta )$.

**Figure 13:**
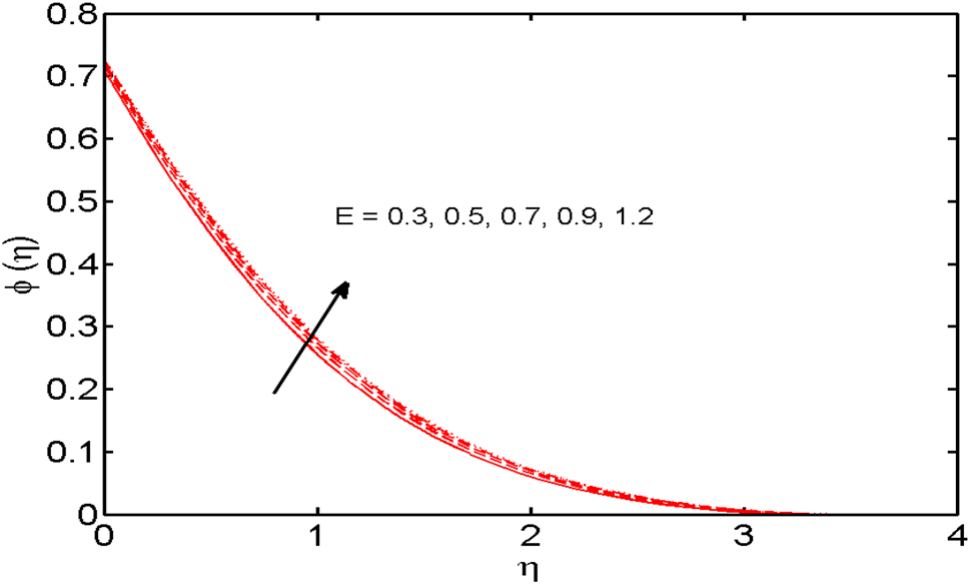
*E* variations vs. $$\phi (\eta )$$.

**Figure 14:**
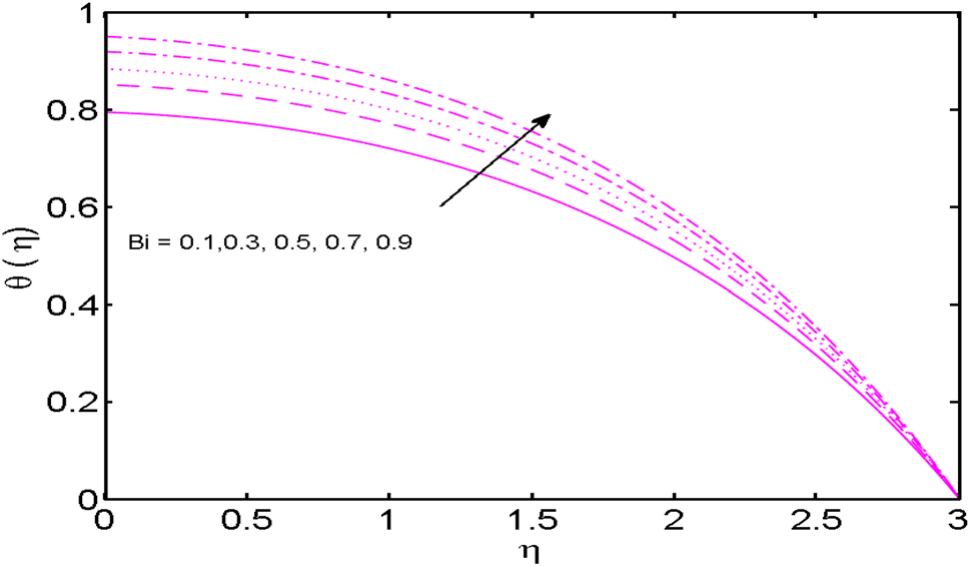
*Bi* variations vs. $\theta (\eta )$.

This segment (Figs. 2, 3, 4, 5, 6, 7, 8, 9, 10, 11, 12, 13, 14) is designed to discuss the salient features of the prominent arising parameters versus the allied profiles. The admissible ranges of the parameters are taken where the resolution of the graphs is best suited. These are $$0.0 \le M \le 1.6,0.0 \le We \le 1.5,0.0 \le \beta_{e} \le 1.2,$$$$0.2 \le \beta_{i} \le 0.9,0.5 \le \lambda * \le 2.0,0.1 \le \gamma \le 0.4,0.1 \le N_{t} \le 0.8,0.2 \le N_{b} \le 0.9,1.0 \le S_{c} \le 3.0,0.1 \le K_{r} \le 0.7,$$ $$0.2 \le \Omega \le 0.5,0.5 \le P_{e} \le 0.8,0.5 \le L_{b} \le 2.0,0.3 \le E \le 1.2,0.1 \le B_{i} \le 0.9.$$ Figures 2 and 3 are illustrated to witness the behavior of the magnetic parameter $$M$$ and Weissenberg number $$We$$ on the velocity profiles in both directions. It is noticed that the velocity profiles $$f^{\prime}(\eta )$$ and $$g^{\prime}(\eta )$$ are decreased for large estimates of $$M$$ and $$We.$$ The fluid velocity in both directions is reduced owing to strong Lorentz force that hinders the movement of the fluid. The Weissenberg number is the quotient of elastic to the viscous forces^48^. Large estimates of $$We$$ mean the significant upturn in the elastic forces. Thus, increasing the relaxation time and eventually, decreased velocity in both directions is witnessed.

The effects of the Hall parameter $\beta_{e} ( = \omega_{e} \tau_{e} ),$ and slip parameter $\beta_{i} ( = \omega_{i} \tau_{i} ),$ on the velocity profiles in both $x$*-* and $y$-directions are depicted in Figs. 4 and 5 respectively. As the Hall parameter $\beta_{e} ( = \omega_{e} \tau_{e} ),$ is defined as the product of electrons’ frequency and electrons’ collisions time. An upsurge in $\beta_{e}$ means either of electrons’ frequency or electron collisions time is escalated or values of both are augmented. It is also verified truth that magnetic and the Hall forces are opposite to each other. As discussed above that magnetic forces oppose the fluid motion. Thus, following the definition, the Hall effect assists the fluid velocity. Similar behavior is comprehended for slip parameter $\beta_{i} ( = \omega_{i} \tau_{i} ),$ in $x$*-* and $y$-directions (Fig. 5). Figure 6 reveals that the variation of the velocity profile for changed estimation of the porosity parameter. It is witnessed that for higher values of the porosity parameter, the velocity profile declines.

In Fig. 7, temperature behavior for varied estimates of the magnetic parameter $M$ and thermal relaxation parameter $\gamma$ is depicted. From the figure, it is seen that the temperature profile is a declining function of respective relaxation time. Higher estimates of relaxation time result in the insulating behavior of the material which is accountable for a decrease in fluid temperature. However, the opposite trend is perceived in the case of a magnetic parameter. Here, for growing values of $M,$ the temperature of the fluid is on rising owing to a thickening effect on the thermal boundary layer which is the result of a strong magnetic field.

The effects of the Brownian motion parameter $N_{b}$ and the Thermophoresis parameter $N_{t}$ on the temperature profile are depicted in Fig. 8. The higher temperature is noticed for greater estimation of both $N_{b}$ and $N_{t}$. This is because the random motion of fluid particles generates more heat and as a result temperature distribution is increased. Similarly, it is observed that the growing estimation of $N_{t}$ boost up the temperature profile. This is because of enhancement in $N_{t}$ push the nanoparticles of fluid away from the hot surface which causes enhancement in temperature profiles.

The impact of the same parameters *i.e.,* Brownian motion parameter $N_{b}$ and the Thermophoresis parameter $N_{t}$ on the concentration distribution are displayed in Fig. 9. It is comprehended that the Brownian motion parameter and the Thermophoresis parameter have escalating and diminishing effects on the concentration profile respectively. Large Brownian motion means the more random motion of fluid particles thus strengthening the concentration of the fluid and an opposing impact is visualized for the thermophoretic parameter on the fluid’s concentration. All these findings are according to the recently published articles^12,49,50^.

Figure 10 is outlined to learn the influence of Schmidt number $Sc$ against the concentration field. For higher estimates of $Sc$ anemic concentration is noted. Schmidt number is in inverse proportionate to Brownian diffusivity. Mounting estimates of $Sc$ results in lowering the Brownian diffusivity. Eventually, this feeble Brownian diffusivity diminishes the concentration profile.

The impact of the chemical reaction parameter $K_{r}$ on the concentration field is witnessed in Fig. 11. It is noted that nanoparticles volume concentration diminishes for large estimates of $K_{r} > 0,$ i.e., destructive case. For growing values of $K_{r} > 0,$ the chemical reaction in case of the destructive case creates much disturbance as compared to the generative case. Therefore, the molecular movement $K_{r} > 0$ is much larger and creates an escalation in mass transport. Eventually, nanoparticles volume concentration decreases.

The variation of Peclet number $Pe,\,$ Bio convection Lewis number $Lb,$ and the Microorganisms concentration difference parameter $\Omega$ on motile density profile is demonstrated in Fig. 12. It is noted that the motile density profile decreases for large estimates of the Bio-convection Peclet number. A rise in $Pe$ results in a decline in the diffusivity of microorganisms and thus the motile density of liquid falls. Figure 10 also highlights the influence of Microorganisms concentration difference parameter and bio-convection Lewis number on motile density profile. It is observed that the rise in $\Omega$ improves the concentration of microorganisms in the ambient boundary layer and reduces the density profile. Also, for growing values of $Lb,$ the diffusivity of microorganisms declines and therefore the motile density decays.

The role of the activation energy parameter $E$ versus the concentration profile is portrayed in Fig. 13. It is comprehended that the concentration is strengthened when estimates of $E$ are enhanced. The generative chemical reaction is boosted when values of $E$ are enhanced. Thus, improvement in concentration is monitored.

The conduct of the Biot number $Bi$ on temperature distribution is examined in Fig. 14. It is seen that, temperature and thickness of the thermal boundary layer upsurge for higher estimates of $Bi$. Here, the expansion in $Bi$ instigates the heat transfer coefficient which pushes more heat from the surface to the fluid. This outcome is consistent with the published result^38^. In this way, the temperature is enhanced. It is also noticed that $Bi = 0$ is associated with an insulated wall while $Bi \to \infty$ act as the constant wall temperature. Higher estimations of $Bi$ turn out as a higher rate of heat transfer. So, $Bi$ can be adopted as a cooling operator in the advanced procedures.

Tables 1 and 2 represent the numerical outcomes of the surface drag force coefficients in both directions and rate of mass flux for different estimates of emerging parameters Hall parameter $\beta_{e} ,$ ion slip parameter $\beta_{i} ,$ magnetic parameter $M,$ Prandtl number $\Pr ,$ Eckert number $Ec,$ and Weissenberg number $We.$ It is witnessed that surface drag force coefficient in $x$-direction decrease when Hall parameter, Weissenberg number, are increased and an opposite tendency is noted for the magnetic parameter, and ion slip parameter. The surface drag force coefficient in $y$-direction is increased for mounting estimates of the magnetic parameter, and an opposing tendency is perceived for growing values of slip and the Hall parameters. However, Table 2 depicts the variations in the rate of mass flux for varied values of $E,K,$ and $N_{t} .$ It is comprehended that for the higher estimates of $K$ and $N_{t} ,$ rate of mass flux escalate and an opposing impact is seen for the values of $E.$ Table 3 reveals the behavior of several parameters on the local density number of motile microorganisms. It is examined that for higher estimates of *Pe* and $\Omega ,$ the local density number of motile microorganisms’ upsurges while decreases for higher *Lb* number.

**Table 1:** Numerical values of Drag force $C_{f} {\text{Re}}_{x}^{1/2}$ against the varied values of the rising parameter.

$M$	$Ec$	$\Pr$	$We$	$\beta_{e}$	$\beta_{i}$	$- \sqrt {\text{Re}} C_{fx}$	$- \sqrt {\text{Re}} C_{gy}$
0	3	3	1	1.2	0.9	1.3748921	0.70471084
0.5						1.3891760	0.72841397
0.7						1.4028943	0.75088802
0.9						1.4211882	0.78043358
0.5	1					1.3891760	0.72841397
	2					1.3891760	0.72841397
	3					1.3891760	0.72841397
	4					1.3891760	0.72841397
	3	1				1.3891764	0.72841397
		2				1.3891764	0.72841397
		3				1.3891764	0.72841397
		4				1.3891764	0.72841397
		3	1			1.389176	0.72841397
			2			1.3117802	0.70827679
			3			1.2240033	0.68654032
			4			0.78790504	0.67413247
			1	0.6		1.4005879	0.73505713
				1		1.3918829	0.73046773
				1.5		1.3862657	0.72576016
				2.1		1.3827605	0.72175107
				1.2	0.2	1.3861825	0.74221885
					0.5	1.3885132	0.73530222
					0.7	1.3890701	0.73155041
					0.9	1.3891760	0.72841397

**Table 2:** Numerical values for $Sh_{x} {\text{Re}}^{ - 0.5}$ against the different estimations of the rising parameter.

*E*	*K*	$N_{t}$	$Sh_{x} {\text{Re}}^{ - 0.5}$
0.4	0.3	0.01	1.396003486334218
0.5			1.382339484071596
0.6			1.369614279513656
0.4	0.4		1.453495332636076
	0.5		1.547835760678850
	0.6		1.720996325877767
	0.3	0.02	1.398055545440856
		0.03	1.400127349140989
		0.04	1.402218982444583

**Table 3:** Numerical values for $Nn_{x} {\text{Re}}_{x}^{ - 1/2}$ against the different values of the rising parameter.

*Lb*	*Pe*	$\Omega$	$Nn_{x} {\text{Re}}_{x}^{ - 1/2}$
0.4	0.3	0.2	1.337005640792250
0.5			1.329845324236785
0.6			1.322647053220159
0.4	0.4		1.426874259673157
	0.5		1.519142631773676
	0.6		1.613768570127874
	0.3	0.2	1.337005640792250
		0.3	1.344975671529449
		0.4	1.352945702320629

The presented model is compared with Wang^51^ (Table 4) in limiting the case. An excellent correlation between the two results is found. The particularly, partially ionized assumption is set to zero. Moreover, the assumption of Bioconvection is also reduced to zero. Thus, making the referred problem similar to the given model here, all extra assumptions are discarded. On comparing the values of $f^{\prime\prime}(0),g^{\prime\prime}(0),f(\infty ),$ and $g(\infty )$ for varied values of stretching ratio parameter $\lambda .$ An outstanding correlation between the two results is accomplished.

**Table 4:** Comparison between values of $f^{\prime\prime}(0),g^{\prime\prime}(0),f(\infty ),$ and $g(\infty )$ with Wang^51^ for numerical values for $\lambda .$

$\lambda$	^51^	Present	^51^	Present	^51^	Present	^51^	Present
	$f^{\prime\prime}(0)$	$f^{\prime\prime}(0)$	$g^{\prime\prime}(0)$	$g^{\prime\prime}(0)$	$f(\infty )$	$f(\infty )$	$g(\infty )$	$g(\infty )$
$0$	$- 1$	$- 1$	$0$	$0$	$1$	$1$	$0$	$0$
$0.25$	$- 1.048813$	$- 1.048793$	$- 0.194564$	$- 0.194543$	$0.907075$	$0.907053$	$0.257986$	$0.257981$
$0.50$	$- 1.093097$	$- 1.093081$	$- 0.465205$	$- 0.465187$	$0.842360$	$0.842323$	$0.451671$	$0.451623$
$0.75$	$- 1.134485$	$- 1.134456$	$- 0.794622$	$- 0.794602$	$0.792308$	$0.792293$	$0.612049$	$0.612021$
$1.00$	$- 1.173720$	$- 1.173698$	$- 1.173720$	$- 1.173711$	$0.751527$	$0.751502$	$0.751527$	$0.751511$

## Final remarks

In this article, steady 3D Tangent hyperbolic nanofluid flow with Hall and Ion slip over a linear bi-directional stretchable surface with Cattaneo–Christov heat flux in a Darcy–Forchheimer permeable medium had been analyzed. Additional effects of heat generation/absorption, bioconvective gyrotactic microorganisms, and chemical reaction with activation energy are also considered. The partially ionized Tangent hyperbolic nanofluid flow amalgamated with chemical reaction with activation energy. The slip and convective conditions are taken at the boundary. The numerical solution of the problem is achieved by using the bvp4c MATLAB technique. The fluid model is unique in its way and has not been discussed in the literature yet. The significant observations (answer to the aforementioned questions) of the model are appended as follows:

- The velocity of the liquid is increased for sizeable values of the ion slip parameter.
- The concentration is strengthened when the values of the activation energy parameter are enhanced.
- The motile density profile is a diminishing function of Peclet and Bioconvection Lewis numbers. Bioconvection microorganisms possess numerous applications in varied engineering disciplines including soil, genetic, wastewater, and microbial, etc.
- The temperature of the liquid augments for the strong magnetic field.
- The velocity profiles are the diminishing function of the magnetic parameter.
- The temperature is decreased for high thermal relaxation parameter values.
- The temperature profile shows dominance for large estimates of Brownian motion and thermophoresis parameters.

## Future possibilities

The envisaged mathematical model may be extended in the following ways:

- The effects of homogeneous–heterogeneous reactions may be considered by replacing the bio-convective relation.
- Some other non-Newtonian fluid may also be considered for the Tangent hyperbolic fluid.
- The Buongiorno model may be replaced by Tiwari and Das model.
- In Tiwari and Das model numerous combinations of nanoparticles and base fluids may be considered.
- The geometry of the problem may also be changed.

## List of symbols

*a, b*: Dimensional constant
*Pr*: Prandtl number
λ*: Porosity parameter
Γ: Transient material constant
*k*: Thermal conductivity of nanofluid
*β*_*i*_: Ion slip parameter
*Q*: Heat generation parameter
*τ*_*w*_: Drag force coefficient
*Bi*: Biot number
*T*: Liquid temperature
*μ*: Dynamic viscosity of the liquid
*λ*_2_: Thermal relaxation time
*Ec*: Eckert number
*E*: Activation energy parameter
*C*_*w*_: Wall concentration
*B*_0_: Strength of magnetic field
*V*_*w*_: Stretching velocity
*N*_*b*_: Parameter of Brownian motion
*D*_*B*_: Coefficient of Brownian diffusion
*τ*: Surface shear stress
*Sc*: Schmidt number
*T*: Liquid temperature
*M*: Magnetic parameter
*Lb*: Bio convection Lewis number
Sh_*x*_: Sherwood number
*n*: Density motile of microorganisms
B: Slip parameter
*N*_∞_: Ambient concentration of microorganisms
*K*_*r*_: Chemical reaction parameter
*γ*: Thermal relaxation parameter
Ω: Microorganisms concentration difference parameter
*N*_0_: Reference concentration of microorganisms
(*u*, *v*, *w*): Components of velocities
*C*_*fx*_,*C*_*gy*_: Drag force
*F*_*r*_: Inertia coefficient
*σ*: Stefan–Boltzmann constant
*δ*: Fluid parameter
*β*_*e*_: Hall parameter
*Re*_*x*_: Reynolds number
*T*_*w*_: Temperature on wall
*We*: Weissenberg number
*ρ*: Density of fluid
*ν*: Kinematic viscosity
*q*_*m*_: Mass flux
*C*: Liquid concentration
*C*_*f*_: Drag force
*c*_*p*_: Capacity of specific heat
*C*_*∞*_: Fluid concentration
*λ*: Stretching ratio parameter
*N*_*t*_: Parameter of thermophoresis
*Nn*_*x*_: Local density number
*Re*_*x*_: Reynolds number
*D*_*T*_: Coefficient of thermophoretic diffusion
*T*_*∞*_: Diffusive temperature
*K*_*c*_: Coefficient of chemical reaction
*Pe*: Bio-convection Peclet number
*D*_*m*_: Microorganism diffusion coefficient
*W*_*c*_: Maximum cell swimming speed
*q*_*n*_: Wall motile organism flux
*D*_*n*_: Diffusivity of microorganism
*h*_*f*_: Heat transfer coefficient
*E*_*a*_: Coefficient of activation energy
*Q*_0_: Coefficient of heat absorption
*T*_*w*_: Temperature on wall
